# Novel Smartphone Paper Sensor for One Health: Monitoring Free Chlorine in Water and Exhaled Breath Condensate

**DOI:** 10.3390/s26103066

**Published:** 2026-05-12

**Authors:** Caterina Cambrea, Robert Josue Rodriguez Arias, Riccardo Desiderio, Faisal Nazir, Maria Maddalena Calabretta, Elisa Michelini

**Affiliations:** 1 Department of Chemistry “Giacomo Ciamician”, University of Bologna, Via P. Gobetti 85, 40129 Bologna, Italy; caterina.cambrea2@unibo.it (C.C.); robert.rodriguez2@unibo.it (R.J.R.A.); riccardo.desiderio2@unibo.it (R.D.); faisal.nazir2@unibo.it (F.N.); maria.calabretta2@unibo.it (M.M.C.); 2 IRCCS Azienda Ospedaliero-Universitaria di Bologna, 40138 Bologna, Italy

**Keywords:** free chlorine, smartphone-based sensor, colorimetric, one health, field analysis, clinical analysis

## Abstract

Disinfection is essential to ensure safe drinking water and hygienic conditions in environmental, industrial, and clinical settings. However, conventional methods for monitoring free residual chlorine are often laboratory-based and not suited for decentralized analysis. Here, we report a novel paper-based colorimetric biosensing platform that translates the ISO 7393-2 standard, a method based on the reaction of chlorine with N,N-diethyl-p-phenylenediamine (DPD), into a portable and user-friendly format. The proposed device integrates the DPD chemistry within a paper architecture, enabling reagent-free operation at the point of need. The sensor provides a rapid visual readout that is detectable by the naked eye, while quantitative analysis is achieved within 3 min through smartphone-based image acquisition. This work constitutes the first implementation of the ISO standard in a portable paper-based format suitable for both environmental and clinical matrices. The sensor provided a detection limit of 12 μM for sodium hypochlorite and was successfully validated in real samples, including bottled water and exhaled breath condensate, with satisfactory recoveries. Furthermore, the stability of the paper-based sensor was assessed under storage conditions of 4 °C and room temperature (23 °C), demonstrating excellent performance over 30 days in both cases, indicating that refrigeration is not required for maintaining sensor performance.

## 1. Introduction

Chlorination is one of the most widely used methods for disinfecting water to ensure its microbiological safety [1]. The process involves the addition of chlorine or chlorine-based compounds, resulting in the formation of disinfecting agents like hypochlorous acid (HClO) and hypochlorite ions (ClO^−^), in which chlorine is in the +1 oxidation state. These agents are highly effective in killing or inactivating a broad spectrum of pathogens, including bacteria, viruses, and protozoa, which can cause waterborne diseases. Chlorine’s disinfection efficiency is primarily attributed to its ability to disrupt microbial cell membranes, denature proteins, and inhibit metabolic processes. However, chlorination can also lead to the formation of disinfection by-products (DBPs), such as trihalomethanes (THMs) and haloacetic acids (HAAs), which are potentially harmful to human health [2,3]. The formation of DBPs is influenced by the quality of source water, organic matter content, and chlorine dosage. In addition, it has been recently reported that chlorine disinfection may increase antibiotic resistance genes in wastewater treatment plants [4]. Despite these potential risks, chlorination remains the most cost-effective and reliable disinfection method for large-scale water treatment. The World Health Organization (WHO) defined a recommended range of residual free chlorine (0.3–0.5 mg L^−1^) depending on water uses [5,6].

An increasing number of cities and regions are encouraging or requiring on-site wastewater treatment and reuse, leading to the establishment of thousands of on-site water reuse systems worldwide. However, the widespread adoption of these systems is impeded by challenges in consistently ensuring the required level of treatment and safeguarding human health during their operation [7]. To guarantee the quality of reclaimed water, several countries have established legal and regulatory frameworks for water reuse (WRFs). Many of these WRFs adopt a fit-for-purpose approach, categorizing water into various quality classes based on the specific reuse application [8]. In this context, the possibility of performing rapid and on-site analyses to guarantee water quality in accordance with the regulatory framework would be highly relevant.

Several analytical methods, optical and electrochemical sensors have been proposed for detecting free chlorine in water [9,10,11,12,13,14], some of them approved by the EPA (United States Environmental Protection Agency) [15] and others by the International Organization for Standardization (ISO) based on titrimetry and colorimetry [16]. The most used approach involves absorbance measurements of the N,N-diethyl-p-phenylenediamine (DPD) oxidation by free chlorine in water [17]. The mechanism is based on a colorimetric reaction that involves the DPD reagent and free chlorine in an aqueous environment, to produce a free radical, called Würster Dye, responsible for the characteristic pink to magenta coloration visible to the naked eye. The intensity of this color is directly proportional to the concentration of free chlorine present in the sample. A secondary, colorless imine product may also be formed, particularly under strongly oxidizing conditions.

Either expensive instrumentation or trained personnel are needed to perform such methods, preventing their easy employment in field analysis. Successful optical sensing strategies based on chemiluminescence [18], fluorescence [19] and colorimetry [20] have been developed, as well as a huge number of electrochemical sensors [21,22]. In addition, paper-based sensors have blossomed in the past years thanks to their high portability, sustainability, and low cost [23,24,25,26]. For example, there are sensors based on silver nanoparticles [24] or carbon dots [26] which offer high sensitivity; however, they need complex synthesis or sophisticated fluorescence readers. There are also colorimetric biosensors using TMB [23] or syringaldazine [25], but in both cases, the stability of the reagents on paper is limited to drinking water analysis, lacking validation in complex systems/matrices.

In addition to water analysis, the detection of chlorine could be important in the diagnosis of respiratory diseases. Sensors for the detection of biomarkers in breath are emerging as a valuable tool, which could be used for non-invasive diagnostics [27]. It has been reported that significantly increased levels of chlorine are found in exhaled breath condensate of patients with allergies during pollen season compared to post-season [28]. Therefore, a low-cost paper sensor that can be used in combination with a smartphone to provide quantitative results would be highly useful both for water and clinical diagnostics, in line with the One Health approach [29].

**Figure 1:**
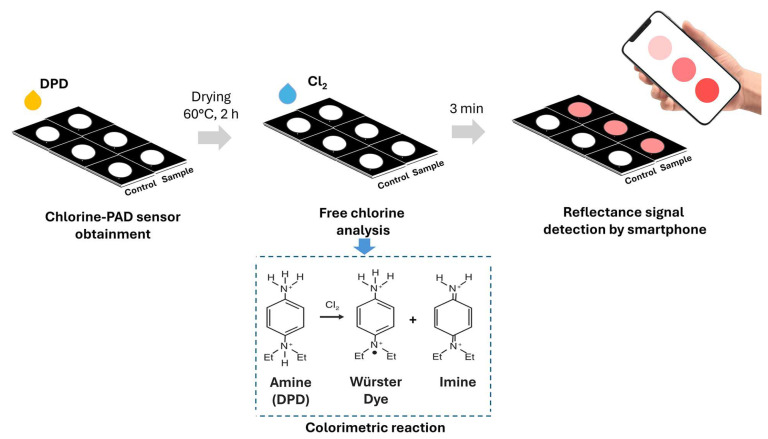
Schematic procedure for the Chlorine PAD obtainment and assay principle of DPD reaction with the optimized configuration. White circles represent the reaction wells delimited by black hydrophobic wax barriers. DPD adsorbed on the hydrophilic paper reacts with free chlorine to produce a pink to magenta dye, with color intensity proportional to the analyte concentration.

In this work, a paper-based colorimetric smartphone sensor overcomes these limitations, like stability of DPD when integrated into cellulose matrix, allowing also a direct quantification of free chlorine in both environmental water and exhaled breath condensate (EBC), offering a low-cost, rapid, portable and user-friendly alternative compared to others. The sensor exploits a colorimetric reaction on paper and allows the detection of free chlorine with the naked eye, providing a simple visual indication. Additionally, quantitative analysis can be performed by capturing images of the sensor using a smartphone camera, allowing for accurate colorimetric detection (Figure 1). This approach provided a detection limit of 12 μM for sodium hypochlorite, making it a promising tool for routine water quality monitoring applications. To assess potential applications both for water monitoring and for the detection of chlorine in clinical samples, the paper sensor was used to detect chlorine in bottled water samples and exhaled breath condensate, showing adequate analytical performance and good recoveries with spiked real samples.

## 2. Materials and Methods

### 2.1. Chemicals, Reagents and Instrumentation

N,N-diethyl-p-phenylenediamine (DPD), NaCl, MgSO_4_, K_2_HPO_4_, K_2_CO_3_, KNO_3_, MgCl_2_, CaCl_2_, FeCl_3_, FeCl_2_, urea, glucose, bovine serum albumin (BSA) and all other chemical reagents were from Sigma-Aldrich (St. Louis, MO, USA). NaClO was from commercially available bleach with a declared NaClO percentage of 3.5% p/p. Chromatography paper (Whatman 1 CHR cellulose) from GE Healthcare (Chicago, IL, USA) was used as support to immobilize the reagents for Chlorine-PAD and the Phaser 8400 office wax printer (Xerox, Norwalk, CT, USA) was used for wax printing. The Honor 200 Pro (Huawei, Shenzhen, China) smartphone camera was used for the acquisition of reflectance signals. The DPD solution was prepared by adding a volume of 55 μL of DPD (97%, d = 0.988 g/mL) in 50 mL of ddH_2_O (with 0.36 M H_2_SO_4_, 0.023 M EDTA) and stored in the dark at 4 °C before use. To test NaClO standard solutions, a dilution buffer (DB) was prepared as follows: 24.0 g di Na_2_HPO_4_, 46.0 g di KH_2_PO_4_, 0.80 g di EDTA, pH 6.8.

### 2.2. Method Optimization on Paper and the Obtainment of the Chlorine-PAD Sensor

First optimizations were focused on obtaining the DPD-chlorine reaction on paper and on defining the optimized ratio of free chlorine and DPD able to react and produce a change in color proportional to the concentration of free chlorine. To this end, a configuration composed of 12 circular areas (3 × 4 array of 6 mm diameter wells) was designed with PowerPoint (Microsoft 365, Microsoft, Redmond, WA, USA) and printed onto Whatman 1 CHR chromatography paper using a wax printer. To create hydrophobic areas, the waxed pattern was heated for 1 min at 100 °C, allowing it to diffuse throughout the paper.

Different volumes of DPD solution and samples composed by different volumetric ratios of NaClO standard solutions and DB (1.0:3.5, 1.0:5.0, 1.0:10.0 and 1.0:20.0) were tested. The DPD immobilization procedure involved the absorption of DPD solution (20 and 40 μL) on paper and its drying at room temperature (23 °C) for 24 h in the dark. Then, a 15 μL volume of the NaClO sample (0.0, 25.0, 35.0 and 50.0 μM) was dispensed on the circular well and incubated at room temperature for 0 to 5 min. Reflectance signals were acquired in standard room lighting, avoiding shadows, with the Honor 200 Pro (Huawei, Shenzhen, China) smartphone placed at a 20 cm vertical distance from the plane of the paper.

Once the optimal DPD volume adsorbed on paper (40 μL) and ratio of sample (1:5.0 of NaClO standard solution: DB) was defined, the drying of DPD solution was also evaluated at 60 °C for 2 h. NaClO calibration curves were obtained by dispensing a 15 μL volume of the NaClO standard solutions (concentration range from 0.0 to 75.0 μM) in triplicate on the circular wells and let to incubate at room temperature for 3 min.

Reflectance measurements were performed using the Honor 200 Pro (Huawei, Shenzhen, China) smartphone placed at a 20 cm vertical distance from the plane of the paper using two different exposure values (EV) of 0 and −1.

### 2.3. Reflectance Signal Acquisition and Data Analysis

Reflectance signal acquisitions were performed with a Honor 200 Pro smartphone (Huawei, Shenzhen, China), equipped with a triple integrated camera, composed of a primary sensor (50MP, f/1.9 aperture lens, OIS, measured 27 mm effective focal length), a secondary ultra-wide sensor (12MP, f/2.2 aperture lens, measured 16 mm effective focal length) and a triple sensor (50MP, f/2.4 aperture lens, OIS). Colorimetric signals were analyzed with ImageJ software (v. 1.53t, National Institutes of Health, Bethesda, MD, USA) as previously described [30] and evaluated by brightness analysis over the circular regions of interest (ROI). GraphPad Prism v.9.4.1 software (GraphPad Software, LaJolla, CA, USA) was used to fit the brightness data related to the concentration to obtain the NaClO calibration curve using second-order polynomial (quadratic) nonlinear regression. The limit of detection (LOD) and limit of quantification (LOQ) were determined according to IUPAC and ICH guidelines [31]. The LOD was calculated as $(y_{blank}-{3\sigma }_{blank}$) where $\sigma_{blank}$ is the standard deviation of the blank. The LOQ was calculated as $(y_{blank}-\;{10\sigma }_{blank}$) where $\sigma_{blank}$ is the standard deviation of the blank. Both these signal values were converted into a concentration unit by using a second-order polynomial regression, where both the interception and the slope of the calibration curve were incorporated. The analyses were performed using three different paper biosensors (well) for each concentration. Additionally, the calibration curves and validation procedure were repeated on three different days to ensure reproducibility.

All the images were acquired indoors under constant artificial lab lighting, avoiding direct sunlight and shadows on the surface of the pads. Each image included the sensing wells and an internal calibration strip with NaClO standards and a blank, and reflectance signals were normalized to the blank before calibration curve fitting.

### 2.4. Selectivity and pH Effect Studies

To evaluate the specificity of the Chlorine-PAD in the presence of potential interferents, selectivity studies were also performed. NaCl, MgCl_2_, CaCl_2_, FeCl_3_, FeCl_2_, K_2_CO_3_, MgSO_4_, K_2_HPO_4_, KNO_3_, BSA, urea and glucose solutions (35 μM) were prepared in ddH_2_O and evaluated individually using the optimized procedure by adding 15 μL of the sample (standard and DB 1:5). After 3 min of incubation time, reflectance signals were acquired with the Honor 200 Pro smartphone camera (EV-0) placed at 20 cm vertical distance from the plane of the paper, in standard room lighting and avoiding shadows. Reflectance signals were compared to those obtained from the Chlorine-PAD sensor tested with the NaClO 35 μM as a positive control (CTR+). pH effects were also evaluated on the Chlorine-PAD sensors using NaClO sample solutions (35 μM) at pH 4.0, 6.5 and 10.0.

### 2.5. Spiked Real Samples for Environmental and Clinical Analysis

The suitability of the Chlorine-PAD to evaluate the free chlorine content in environmental and clinical samples was also evaluated. Bottled water samples (San Benedetto, Acqua Minerale San Benedetto S.p.a., Scorzè, Italy) with a declared chloride concentration of 3.0 mg/L were spiked with NaClO (35 μM). To simulate a clinical sample, exhaled breath condensate was first obtained. To this end, a cooling device was created based on previous studies performed by Khorshid et al. to exhaled breath condensate into a liquid form to obtain the BEC [32]. The principle of this system relies on the decrease in temperature and, to facilitate this process, a metal base and freezer pack were cooled to −20 °C overnight. Additionally, a tube from a sterile IV infusion set was used. The metal base and a polypropylene (PP) tube for sample collection were placed in a cooler, and the freezer pack was arranged around the metal base. Then, the sterile IV infusion tube was positioned with the end part inside the PP tube, and the other part was used for collecting the sample.

NaClO was then added to the sample to reach a concentration of 35 μM, a similar concentration (~30 μM) found in asthmatic patients when exposed to airborne allergens in pollen season [28]. The image was acquired with the Honor 200 Pro smartphone camera, analyzed with ImageJ software and results compared to those obtained with NaClO (35 μM) in ddH_2_O, bottled water and exhaled breath condensates.

### 2.6. Stability Studies

Chlorine-PAD stability studies were also performed by measuring the colorimetric signals of sensing papers stored at +4 °C and at room temperature (23 °C) for 30 days. A total number of 14 Chlorine-PADs were prepared according to the optimized analytical protocol (absorption of 40 μL of DPD solution onto the paper substrate, incubation for 2 h at 60 °C under dark conditions). A total of 7 Chlorine-PADs were stored in the dark at room temperature (23 °C) and the other 7 PADs at +4 °C. Then, the sensor response was assessed by the addition of 15 μL of NaClO (35 μM) in ddH_2_O with an incubation time of 3 min at room temperature (23 °C) at days 0, 1, 7, 14, 21 and 30. Colorimetric signals were acquired with a smartphone camera (Honor 200 Pro) and analyzed with ImageJ software.

## 3. Results

### 3.1. Optimization of Chlorine-PAD Sensor

First optimizations were focused on defining the optimized ratio of free chlorine and DPD able to react and produce a change in color proportional to the concentration of free chlorine. This was necessary to avoid secondary oxidation of the Würster dye that causes a colorless imine product responsible for the bleaching effect when chlorine levels exceed the range of the DPD. This implies that the DPD method cannot be used in detecting high concentrations of free chlorine. In addition, the use of the DB solution is essential to satisfy minimum requirements as the pH adjustment of the sample in the pH range typically from 6.2 to 6.5.

Different volumes of DPD (20 and 40 μL) and different ratios (1.0:3.5; 1.0:5.0, 1.0:10.0 and 1.0:20.0) of NaClO standard solution in DB solution (total volume of 15 μL/well) have been assessed to identify the condition providing the highest sensitivity in the detection of free chlorine. NaClO is widely used as a broad-spectrum disinfectant with effective activity on viruses, bacteria, fungi, and mycobacteria. This contributes to an increase in the free chlorine content in water, leading to the accumulation of potential carcinogenic compounds [33].

Starting from preliminary experiments carried out with a Chlorine-PAD sensor obtained by deposition of 20 μL volume of DPD (drying at 23 °C for 24 h), reflectance signals were acquired with Honor Pro 200 smartphone camera after addition of 15 μL of NaClO standard solutions (25.0, 35.0 and 50.0 μM) at different incubation times (from 0 to 5 min). As shown in Supplementary Materials (Figure S1), the presence of free chlorine led to a change in color visible to the naked eye proportional to the concentration, from white to pinkish/red color immediately after NaClO deposition (0 min). However, with increasing the incubation time, no differences in reflectance signals were obtained with 25.0 and 35.0 μM of NaClO, while a bleaching effect at the highest tested concentration (50.0 μM) was observed. For this reason, to avoid the concentration range of NaClO exceeding the range of the DPD, other experiments were carried out testing 15 μL of NaClO standard solutions in DB with the ratio of 1.0:5.0 (Supplementary Materials, Figure S2), ratio of 1.0:10.0 (Supplementary Materials, Figure S3) and ratio of 1.0:20.0 (Supplementary Materials, Figure S4). Despite a change in color being obtained for the three NaClO standard solutions tested (ratio NaClO in DB of 1.0:5.0), no significant differences proportional to the concentration were observed (Supplementary Materials, Figure S2). Further increasing the ratio between NaClO and DB (1.0: 10.0), the change in colors resulted in a milder (Supplementary Materials, Figure S3) to being invisible (Supplementary Materials, Figure S4). The same experimental session procedure was also evaluated with the Chlorine-PAD sensor obtained by deposition of an increased volume of DPD (40 μL, drying at 23 °C for 24 h). Also, in this case, the presence of free chlorine led to a change in color after the addition of 15 μL of NaClO standard solutions in DB (ratio of 1.0:3.5) proportionally to the tested concentrations (25.0, 35.0 and 50.0 μM), but the bleaching effect was immediately visible after the addition of 50.0 μM NaClO standard solution (Supplementary Materials, Figure S5). By increasing the ratio between NaClO in DB solution (1.0:5.0), the bleaching effect was avoided at 50.0 μM of NaClO and a significant difference in reflectance signals, at varying concentrations visible by naked eye, was obtained (Supplementary Materials, Figure S6), confirming its applicability in the concentration range of interest for environmental and clinical samples.

Conversely, further increasing the dilution ratio between the NaClO standard and the DB solution to 1:10 (Supplementary Materials, Figure S7) and 1:20 (Supplementary Materials, Figure S8) resulted in a decrease in reflectance intensity. This behavior can likely be attributed to the reduced DPD concentration, which becomes insufficient to fully react with free chlorine and generate a measurable colorimetric response.

### 3.2. Free Chlorine Calibration Curves Optimization and Reflectance Measurements

Once the DPD volume adsorbed on paper and the optimal ratio between the NaClO and DB solution (1:5) was defined, NaClO calibration curves (from 0.0 to 75.0 μM) were obtained with 3 min of incubation at room temperature, measuring reflectance signals with the Honor Pro 200 smartphone camera with EV of either 0 or −1. This work concentration range was selected to cover maximum residual free chlorine concentrations defined by the WHO (0.3–0.5 mg L^−1^, corresponding to 4.2–7.1 μM) depending on the water uses. In addition, this range also covers concentrations potentially useful for diagnostics of respiratory diseases, for example, found in exhaled breath condensate of patients with allergy [28]. As shown in Figure S9, using the EV of −1, an increased variability proportional to the concentration was observed, with a coefficient of variation (CV%) between 4 and 14%. Reflectance signals were also acquired with the EV of zero, obtaining a reduction in variability between replicates (CV% ~ 2–3%) and a nonlinear NaClO calibration curve was obtained with a coefficient of determination (R^2^) of 0.9593. These results could be related to the degradation of DPD reagent in air when the absorption method is performed for 24 h at 23 °C, preventing the LODs and LOQs from being obtained.

**Figure 2:**
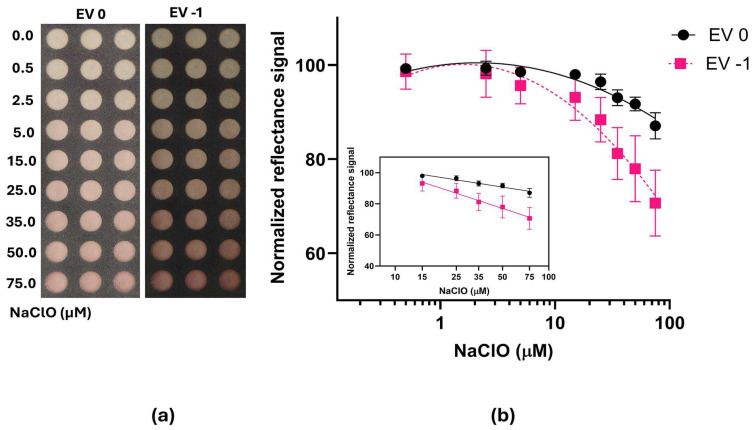
NaClO calibration curves obtained with the Chlorine-PAD sensor after 3 min of incubation time. (**a**) Colorimetric images of the Chlorine-PAD sensor acquired with the Honor Pro 200 smartphone camera (EV 0 and EV −1); (**b**) graphical elaboration of reflectance signals analyzed with ImageJ software. The inset represents the linear correlation in the higher range of the signal response vs. NaClO concentrations.

To increase the sensitivity of the Chlorine-PAD, the time and the temperature of the DPD absorption (60 °C, 2 h) on paper were further investigated. NaClO calibration curves were obtained with the same procedure described previously and imaged with the Honor Pro 200 smartphone camera (EV of 0 and −1). Despite the difference in colorimetric signals related to the NaClO concentration appearing more pronounced in the picture taken with EV −1 than that with EV 0 (Figure 2a), higher variability between replicates was found with the analysis with ImageJ software (Figure 2b). This allowed us to obtain comparable LODs of about 11.4 and 12.0 μM, which were about two orders of magnitude higher than obtained with another smartphone-based colorimetric device (0.23 μM) but with higher simplicity of use [5]. However, despite the advantages related to the sensitivity and portability for field analysis, the structure of the reported device involves the use of several additional components like a mini-flashlight, test solution reservoir, inlet of colorimetric tube, light shielding box and optical filter array. LOQs of about 37.8 and 62.4 μM were also obtained for pictures taken with EV 0 and EV −1, respectively, confirming that the reflectance acquisition by smartphone camera with EV of zero provides accurate quantification of free chlorine in the concentration range of interest for water and clinical analysis.

To address the issue of data reproducibility when using different smartphones or CMOS cameras and different light conditions, each PAD was designed to include an internal calibration curve with NaClO standards at different concentrations and a blank well. The reflectance signal of each well is normalized by dividing it by the signal of the blank well. These normalized signals are used to build a regression model, which is then used to interpolate the concentration of unknown samples. This on-board calibration compensates for variability between different smartphone cameras and minor changes in lighting conditions, provided that all the wells on the PAD are uniformly illuminated.

### 3.3. Selectivity Studies and pH Effects

In the optimized analytical protocol, the fabrication of the Chlorine-PAD involved the absorption of 40 μL of DPD solution onto the paper substrate, followed by incubation for 2 h at 60 °C under dark conditions. Subsequently, the sample diluted in DB solution (pH 6.5) at a 1.0:5.0 ratio was applied for analysis.

**Figure 3:**
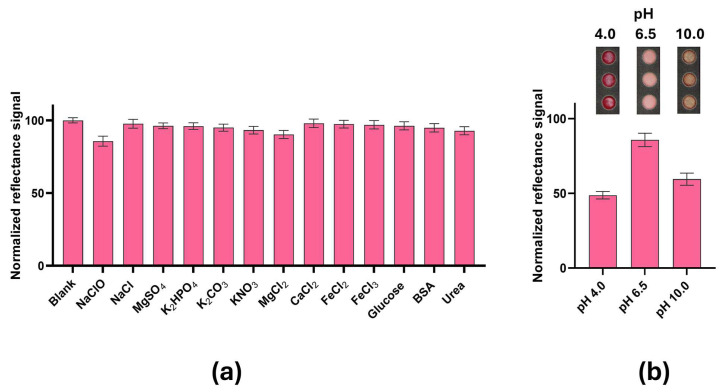
Analytical performance of the Chlorine–PAD. (**a**) Selectivity studies of the Chlorine-PAD with different interferents (35 μM) compared to NaClO 35 μM (CTR+). Reflectance signals are normalized to the blank (ddH_2_O); (**b**) pH effects on the Chlorine-PAD.

The selectivity of the Chlorine-PAD was evaluated to assess the potential activity of common interferents in water and clinical samples, including ions (MgCl_2_, CaCl_2_, FeCl_3_, FeCl_2_, NaCl, MgSO_4_, K_2_HPO_4_, K_2_CO_3_, KNO_3_) and compounds like urea, glucose and BSA. All interferents were tested at 35 μM, a mid-range concentration matching NaClO standards and environmentally/clinically relevant levels. All solutions were prepared in ddH_2_O and analyzed with the optimized protocol. Reflectance measurements were performed with the Honor Pro 200 smartphone camera. Results were compared to NaClO (35 μM) used as CTR (+) and normalized with respect to the ddH_2_O solution (blank). As shown in Figure 3a, no variation in reflectance signals was found for the tested compounds (Figure S10), confirming that potential interferents do not affect the specificity of the Chlorine-PAD sensor to free chlorine detection. This good selectivity prompts further use for water analyses, and satisfies the ASSURED (Affordable, Sensitive, Specific, User-friendly, Rapid and Robust, Equipment-free and Deliverable to end users) set by the WHO [34].

The effect of pH was also evaluated with NaClO solutions (35 μM) at pH 4.0, 6.5 and 10.0 (Figure 3b). Decreased reflectance signals of about 51 ± 5%, 15 ± 5% and 41 ± 6% were observed at pH 4.0, 6.5 and 10.0, respectively. This is most likely due to NaClO, which is predominantly present in acid conditions in the protonated form, being more oxidant than its anion form.

### 3.4. Real Sample Analysis and Chlorine-PAD Storage Stability

**Figure 4:**
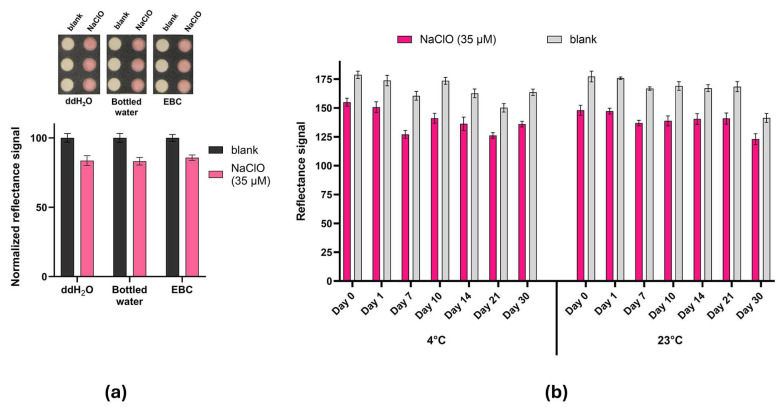
(**a**) Real sample analysis obtained with the Chlorine-PAD, by spiking NaClO (35μM) in ddH_2_O, commercial bottled water, and exhaled breath condensate (EBC); (**b**) stability of the Chlorine-PAD stored at +4 °C and at room temperature (23 °C).

The analytical performance of the Chlorine-PAD was evaluated with environmental and clinical real samples, including commercial bottled water and exhaled breath condensate. Samples were spiked with 35 μM NaClO (Figure 4a). Exhaled breath condensate from a healthy volunteer was chosen to simulate a biological matrix spiked with NaClO 35 μM to simulate a clinical sample of an asthmatic patient. In fact, a previous study revealed that chlorine (Cl_2_) concentrations in exhaled breath condensate of asthmatic patients are significantly higher during the pollen season compared to periods without airborne allergens [28]. Reflectance measurements were performed with the Honor Pro 200 smartphone camera, and results were normalized to the blank (either ddH_2_O, bottled water or exhaled breath condensate). Brightness signals in spiked real samples were comparable to NaClO standards.

When compared to previously reported sensors for free chlorine, such as the sensor developed by Yen et al. [35], it must be pointed out that the LOD is higher; however, the fabrication of the chemiresistive sensor is more complex, and it does not provide a visual readout.

Chlorine-PAD storage stability was evaluated during storage at +4 °C and 23 °C, and its analytical performance for the detection of NaClO was assessed by performing the assay after 1, 7, 10, 21, and 30 days, acquiring the colorimetric images and comparing reflectance signals to the values obtained for the freshly prepared Chlorine-PADs (day 0). The assay provided excellent results after 30 days at +4 C° and at 23 °C, showing a responsiveness of 104 ± 3% and of 96 ± 3% compared to the test performed on day 0, demonstrating no need for refrigeration (Figure 4b).

The high level of stability obtained in these experiments demonstrates that the Chlorine-PAD can be used over a long period of time. This feature allows the sensor to be shipped and used in remote rural areas.

Reproducibility was also investigated by testing Chlorine-PADs produced on different dates and tested with a concentration of NaClO (35 μM), demonstrating excellent reproducibility with a relative standard deviation (RSD%) of about 3.7%.

## 4. Conclusions

In this work, for the first time, the development of a colorimetric low-cost paper-based sensor for free chlorine detection, with an estimated cost per sample of less than 0.1 euros, is reported, which is much lower than the cost per assay with a commercial kit, which is approximately five euros (e.g., Merck chlorine colorimetric test kit).

The Chlorine-PAD exploits the capability of DPD to react with residual free chlorine to produce magenta color, visible to the naked eye.

Quantitative analysis can be performed by capturing images of the sensor using a smartphone, allowing for accurate colorimetric detection in only 3 min of incubation time. This approach achieved a detection limit of 12 μM for sodium hypochlorite, making it a promising tool for routine water quality monitoring applications. To assess potential applications both for water monitoring and for the detection of chlorine in clinical samples, the paper sensor was used to detect chlorine in tap water samples and exhaled breath condensate, showing adequate analytical performance with spiked real samples.

## Data Availability

The original contributions presented in this study are included in the article/Supplementary Materials. Further inquiries can be directed to the corresponding author.

## Supplementary Materials

The following supporting information can be downloaded at: https://www.mdpi.com/article/10.3390/s26103066/s1, Figure S1: Chlorine-PAD obtained by drying 20 μL of DPD at room temperature (23 °C) for 24 h in the dark and tested with 15 μL of the sample prepared in a 1.0:3.5 ratio; Figure S2: Chlorine-PAD obtained by drying 20 μL of DPD at room temperature (23 °C) for 24 h in the dark and tested with 15 μL of the sample prepared in a 1.0:5.0 ratio; Figure S3: Chlorine-PAD obtained by drying 20 μL of DPD at room temperature (23 °C) for 24 h in the dark and tested with 15 μL of the sample prepared in a 1.0:10.0 ratio; Figure S4: Chlorine-PAD obtained by drying 20 μL of DPD at room temperature (23 °C) for 24 h in the dark and tested with 15 μL of the sample prepared in a 1.0:20.0 ratio; Figure S5: Chlorine-PAD obtained by drying 40 μL of DPD at room temperature (23 °C) for 24 h in the dark and tested with 15 μL of the sample prepared in a 1.0:3.5 ratio; Figure S6: Chlorine-PAD obtained by drying 40 μL of DPD at room temperature (23 °C) for 24 h in the dark and tested with 15 μL of the sample prepared in a 1.0:5.0 ratio; Figure S7: Chlorine-PAD obtained by drying 40 μL of DPD at room temperature (23 °C) for 24 h in the dark and tested with 15 μL of the sample prepared in a 1.0:10.0 ratio; Figure S8: Chlorine-PAD obtained by drying 40 μL of DPD at room temperature (23 °C) for 24 h in the dark and tested with 15 μL of the sample prepared in a 1.0:20.0 ratio; Figure S9: Image of calibration curve for NaClO obtained using the Chlorine-PAD, obtained by drying 40 μL of DPD at room temperature (23 °C) for 24 h. Figure S10: Selectivity studies of the Chlorine-PAD with different interferents (35 μM) compared to NaClO 35 μM (CTR+).
