# Supplementary material for: Novel Smartphone Paper Sensor for One Health: Monitoring Free Chlorine in Water and Exhaled Breath Condensate
Source: Sensors (Basel). 2026 May 12;26(10):3066. doi: 10.3390/s26103066 (PMC13211164; doi:10.3390/s26103066)
Supplement: Supplementary file 1 [file sensors-26-03066-s001.zip › sensors-4247977-supplementary.pdf]

Supporting Material

Novel Smartphone Paper Sensor for One Health: Monitoring Free Chlorine in Water and Exhaled Breath Condensate

Caterina Cambrea [1,†], Robert Josue Rodriguez Arias [1], Riccardo Desiderio [1], Faisal Nazir [1], Maria Maddalena Calabretta [1,†] and Elisa Michelini [1,2,*]

[1] Department of Chemistry "Giacomo Ciamician", University of Bologna, Via P. Gobetti 85, 40129 Bologna, Italy; caterina.cambrea2@unibo.it (C.C.); robert.rodriguez2@unibo.it (R.J.R.A.); riccardo.desiderio2@unibo.it (R.D.); faisal.nazir2@unibo.it (F.N.); maria.calabretta2@unibo.it (M.M.C.)
[2] IRCCS Azienda Ospedaliero-Universitaria di Bologna, 40138 Bologna, Italy
* Correspondence: elisa.michelini8@unibo.it
† These authors contributed equally to this work.

TABLE OF CONTENTS

Figure S1: Images of the Chlorine-PAD obtained by drying 20 µL of DPD at room temperature (23 °C) for 24 h in the dark and tested with 15 µL of sample prepared in a 1.0:3.5 ratio 2

Figure S2: Images of the Chlorine-PAD obtained by drying 20 µL of DPD at room temperature (23 °C) for 24 h in the dark and tested with 15 µL of sample prepared in a 1.0:5.0 ratio 3

Figure S3: Images of the Chlorine-PAD obtained by drying 20 µL of DPD at room temperature (23 °C) for 24 h in the dark and tested with 15 µL of sample prepared in a 1.0:10.0 ratio r. 4

Figure S4: Images of the Chlorine-PAD obtained by drying 20 µL of DPD at room temperature (23 °C) for 24 h in the dark and tested with 15 µL of sample prepared in a 1.0:20.0 ratio. 5

Figure S5: Images of the Chlorine-PAD obtained by drying 40 µL of DPD at room temperature (23 °C) for 24 h in the dark and tested with 15 µL of sample prepared in a 1.0:3.5 ratio 6

Figure S6: Images of the Chlorine-PAD obtained by drying 40 µL of DPD at room temperature (23 °C) for 24 h in the dark and tested with 15 µL of sample prepared in a 1.0:5.0 ratio 7

Figure S7: Images of the Chlorine-PAD obtained by drying 40 µL of DPD at room temperature (23 °C) for 24 h in the dark and tested with 15 µL of sample prepared in a 1.0:10.0 ratio 8

Figure S8: Images of the Chlorine-PAD obtained by drying 40 µL of DPD at room temperature (23 °C) for 24 h in the dark and tested with 15 µL of sample prepared in a 1.0:20.0 ratio. 9

Figure S9: Calibration curve for NaClO obtained using the Chlorine-PAD, obtained by drying 40 µL of DPD at room temperature (23 °C) for 24 h. The test was performed by adding 15 µL of sample prepared in a 1.0:5.0 ratio..... 10

Figure S10: Selectivity studies of the Chlorine-PAD with different interferents (35 µM) compared to NaClO 35 µM (CTR+). Reflectance signals are normalized to the CTR(-). 11

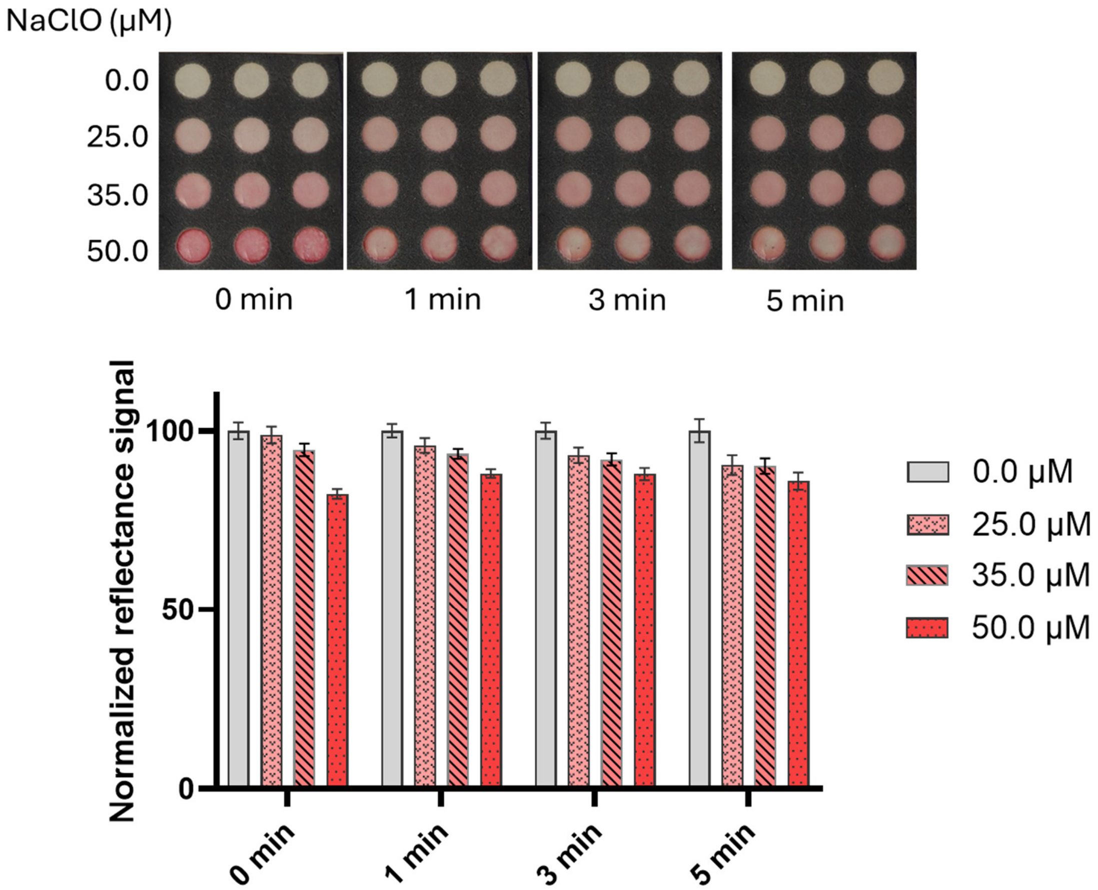

Figure S1: Images of the Chlorine-PAD obtained by drying 20 μL of DPD at room temperature (23 °C) for 24 h in the dark and tested with 15 μL of sample prepared in a 1.0:3.5 ratio (20 μL DB solution + 8 μL NaClO). Reflectance signals were acquired after 0, 1, 3 and 5 minutes in standard room lighting, avoiding shadows, with the Honor 200 PRO smartphone placed at 20 cm vertical distance from the plane of the paper.

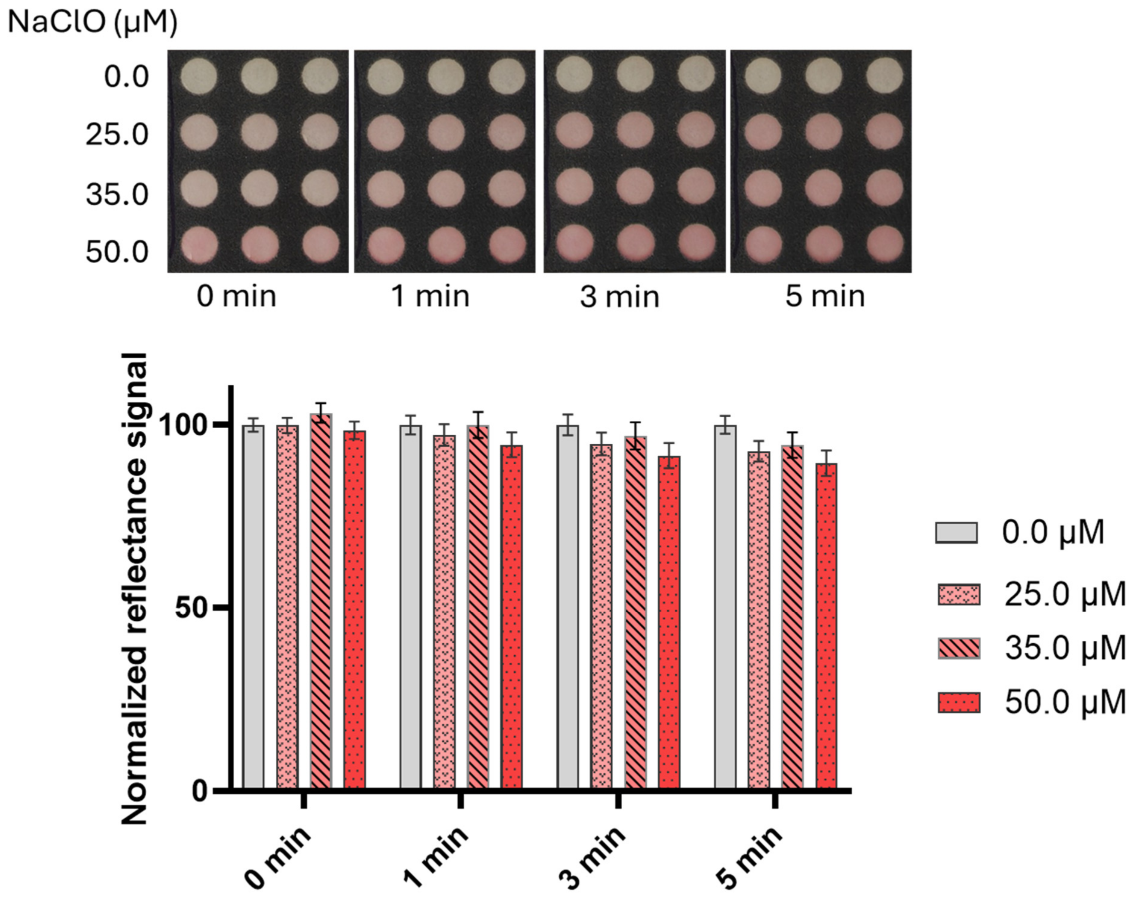

Figure S2: Images of the Chlorine-PAD obtained by drying 20 µL of DPD at room temperature (23 °C) for 24 h in the dark and tested with 15 µL of sample prepared in a 1.0:5.0 ratio (20 µL DB solution + 5 µL NaClO). Reflectance signals were acquired after 0, 1, 3 and 5 minutes in standard room lighting, avoiding shadows, with the Honor 200 PRO smartphone placed at 20 cm vertical distance from the plane of the paper.

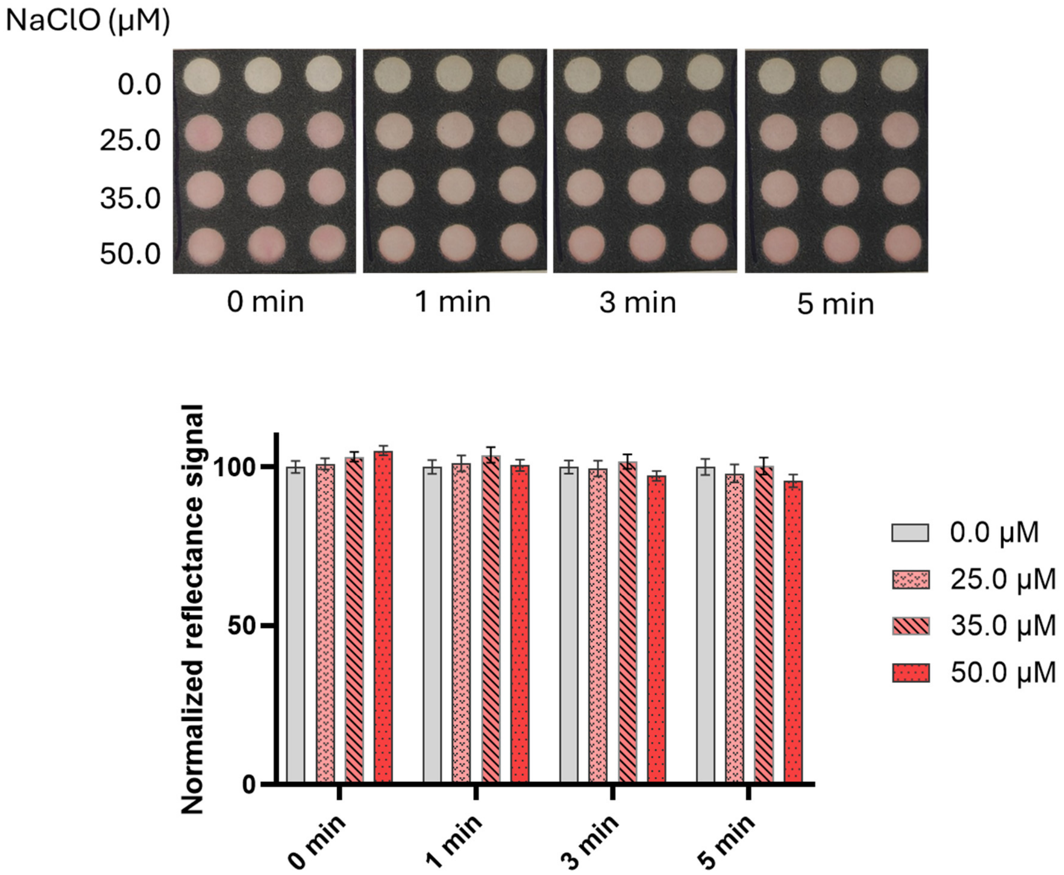

Figure S3: Images of the Chlorine-PAD obtained by drying 20 μL of DPD at room temperature (23 °C) for 24 h in the dark and tested with 15 μL of sample prepared in a 1.0:10.0 ratio (18 μL DB solution + 2 μL NaClO). Reflectance signals were acquired after 0, 1, 3 and 5 minutes in standard room lighting, avoiding shadows, with the Honor 200 PRO smartphone placed at 20 cm vertical distance from the plane of the paper.

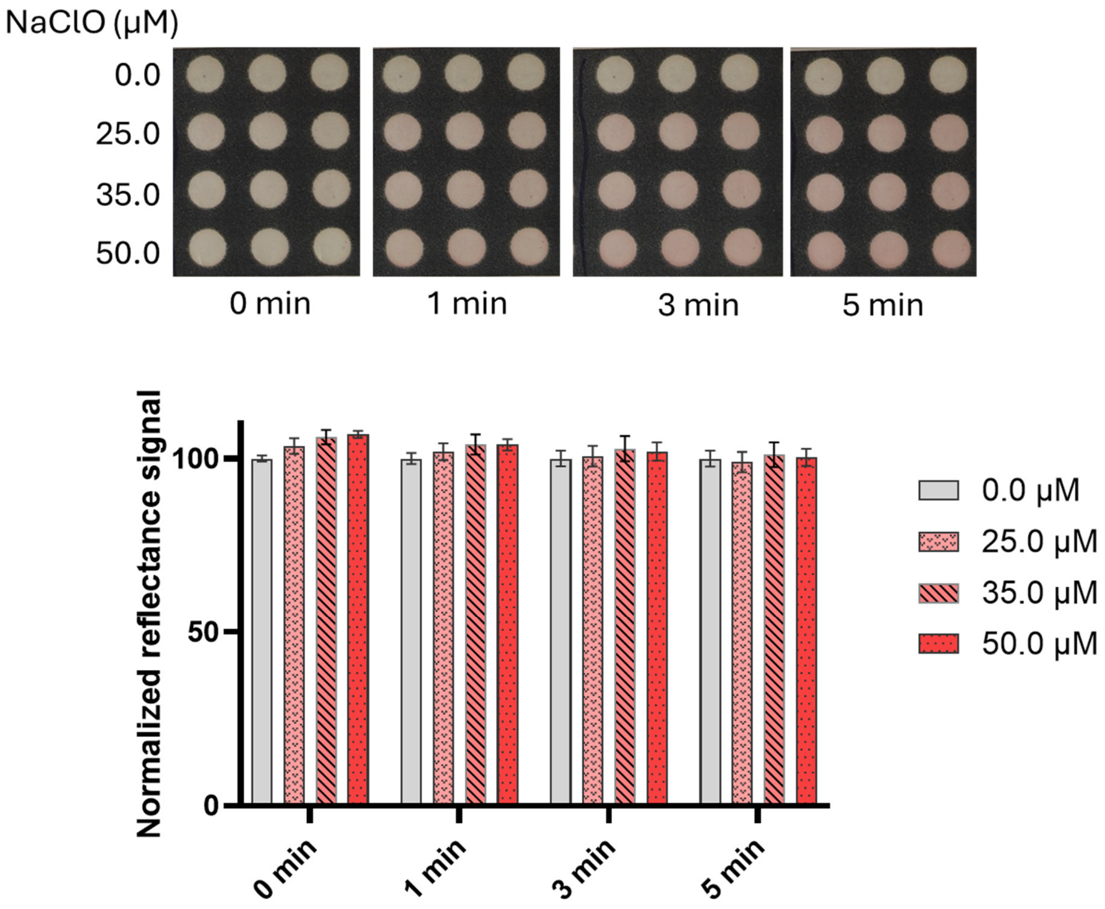

Figure S4: Images of the Chlorine-PAD obtained by drying 20 μL of DPD at room temperature (23 °C) for 24 h in the dark and tested with 15 μL of sample prepared in a 1.0:20.0 ratio (19 μL DB solution + 1 μL NaClO). Reflectance signals were acquired after 0, 1, 3 and 5 minutes in standard room lighting, avoiding shadows, with the Honor 200 PRO smartphone placed at 20 cm vertical distance from the plane of the paper.

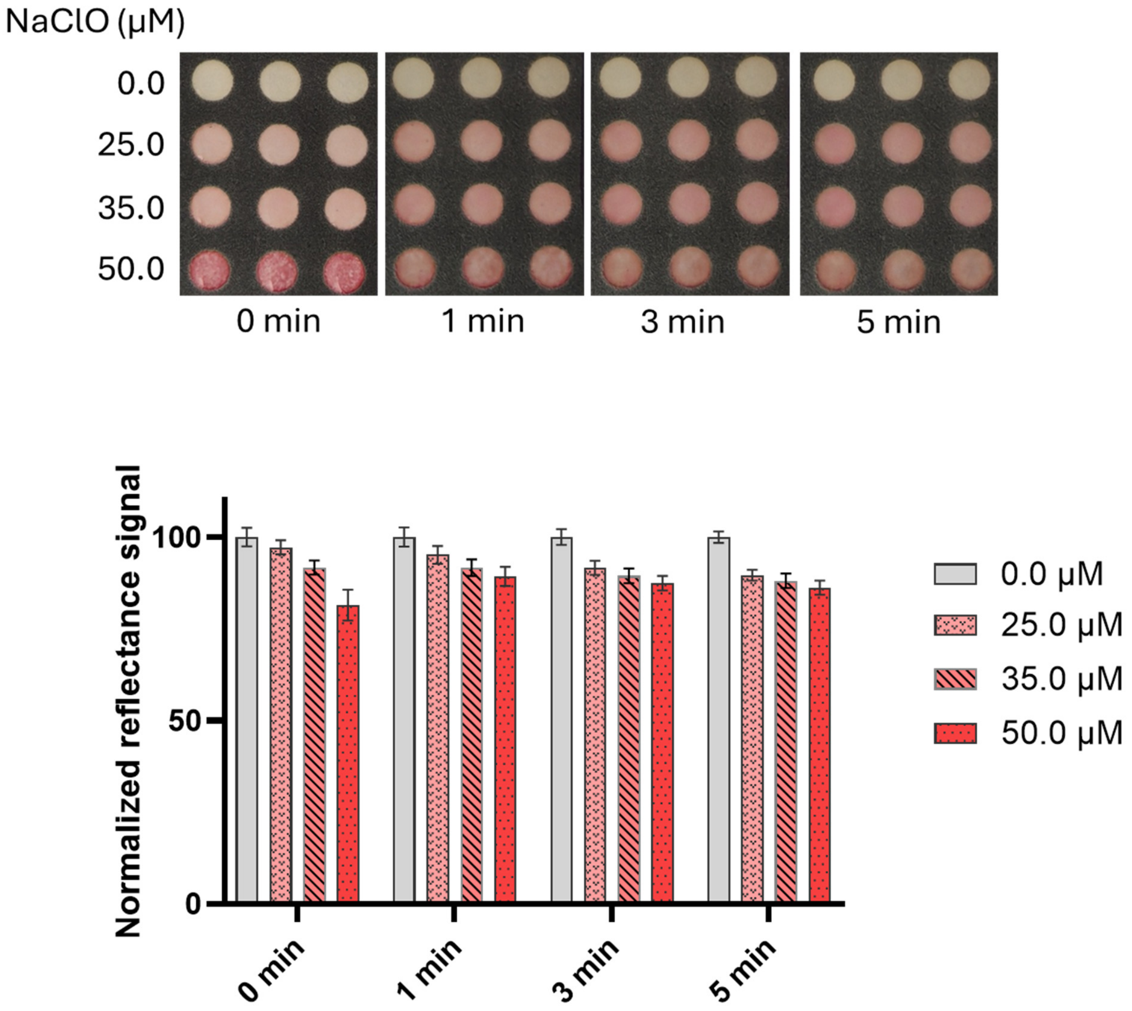

Figure S5: Images of the Chlorine-PAD obtained by drying 40 µL of DPD at room temperature (23 °C) for 24 h in the dark and tested with 15 µL of sample prepared in a 1.0:3.5 ratio (20 µL DB solution + 8 µL NaClO). Reflectance signals were acquired after 0, 1, 3 and 5 minutes in standard room lighting, avoiding shadows, with the Honor 200 PRO smartphone placed at 20 cm vertical distance from the plane of the paper.

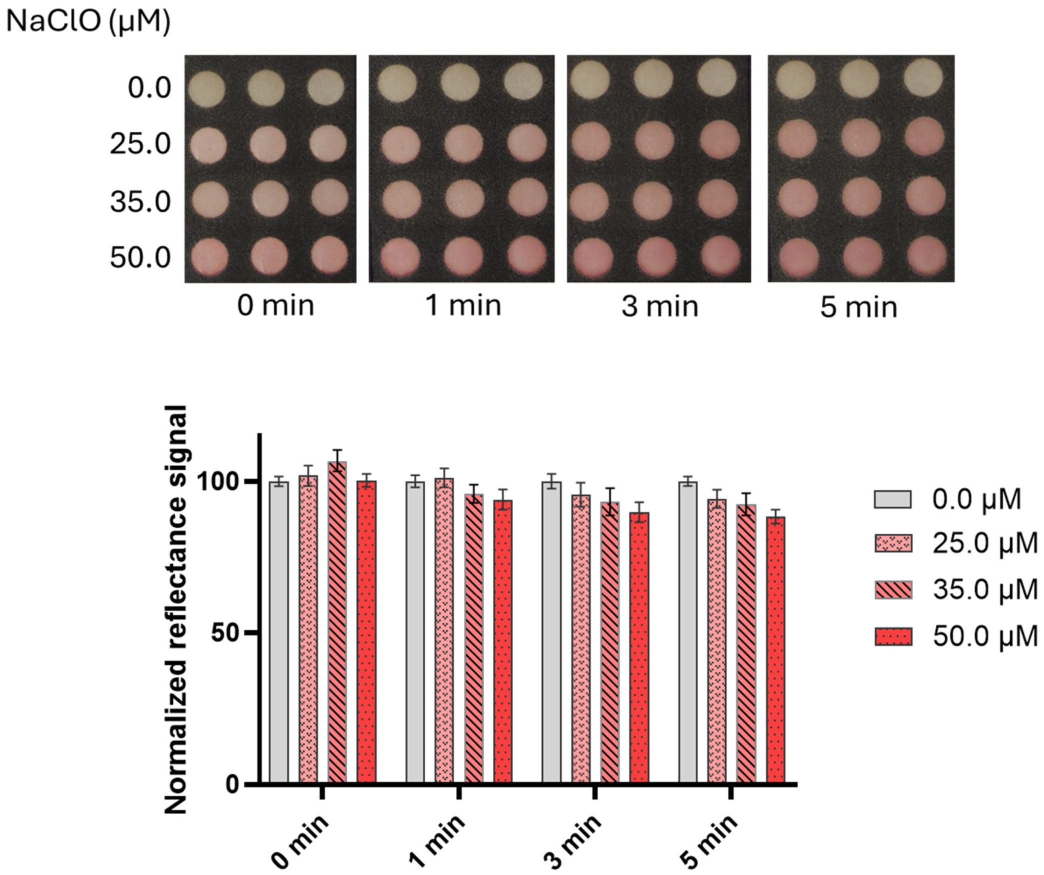

Figure S6: Images of the Chlorine-PAD obtained by drying 40 μL of DPD at room temperature (23 °C) for 24 h in the dark and tested with 15 μL of sample prepared in a 1.0:5.0 ratio (20 μL DB solution + 5 μL NaClO). Reflectance signals were acquired after 0, 1, 3 and 5 minutes in standard room lighting, avoiding shadows, with the Honor 200 PRO smartphone placed at 20 cm vertical distance from the plane of the paper.

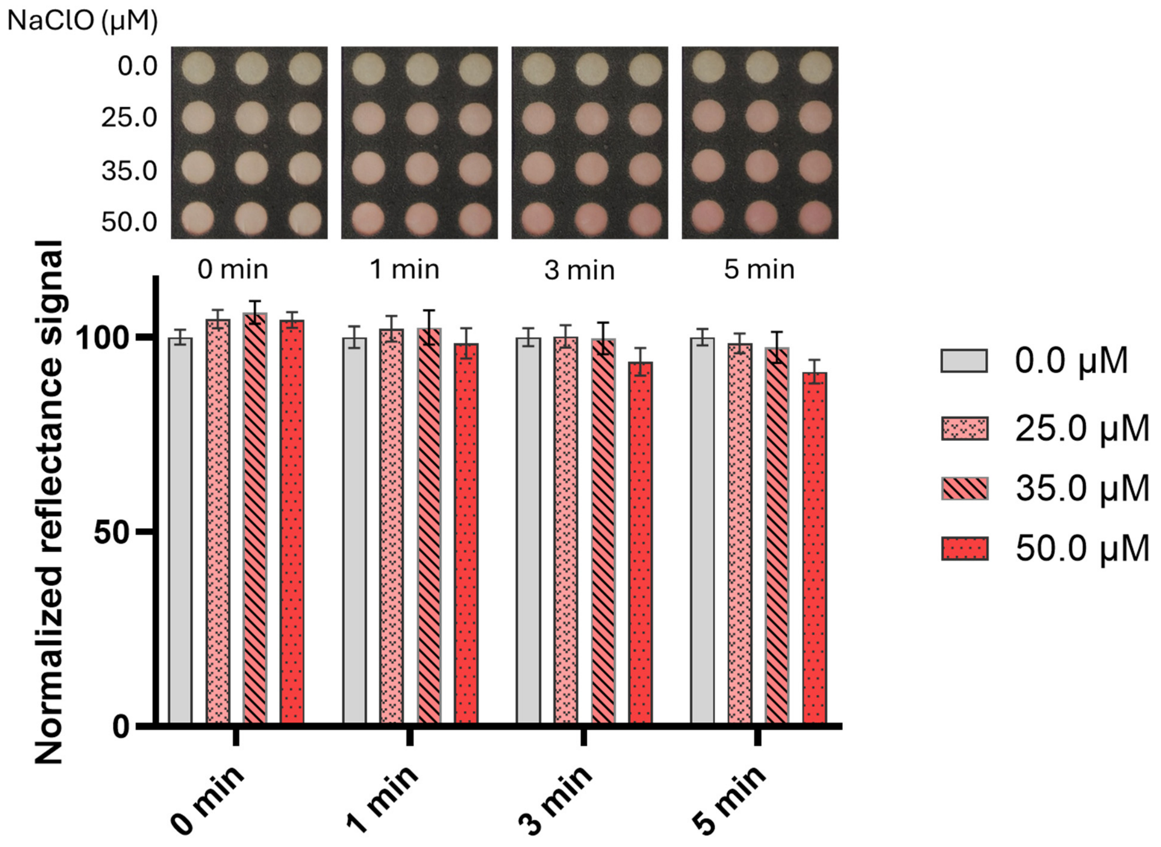

Figure S7: Images of the Chlorine-PAD obtained by drying 40 µL of DPD at room temperature (23 °C) for 24 h in the dark and tested with 15 µL of sample prepared in a 1.0:10.0 ratio (18 µL DB solution + 2 µL NaClO). Reflectance signals were acquired after 0, 1, 3 and 5 minutes in standard room lighting, avoiding shadows, with the Honor 200 PRO smartphone placed at 20 cm vertical distance from the plane of the paper.

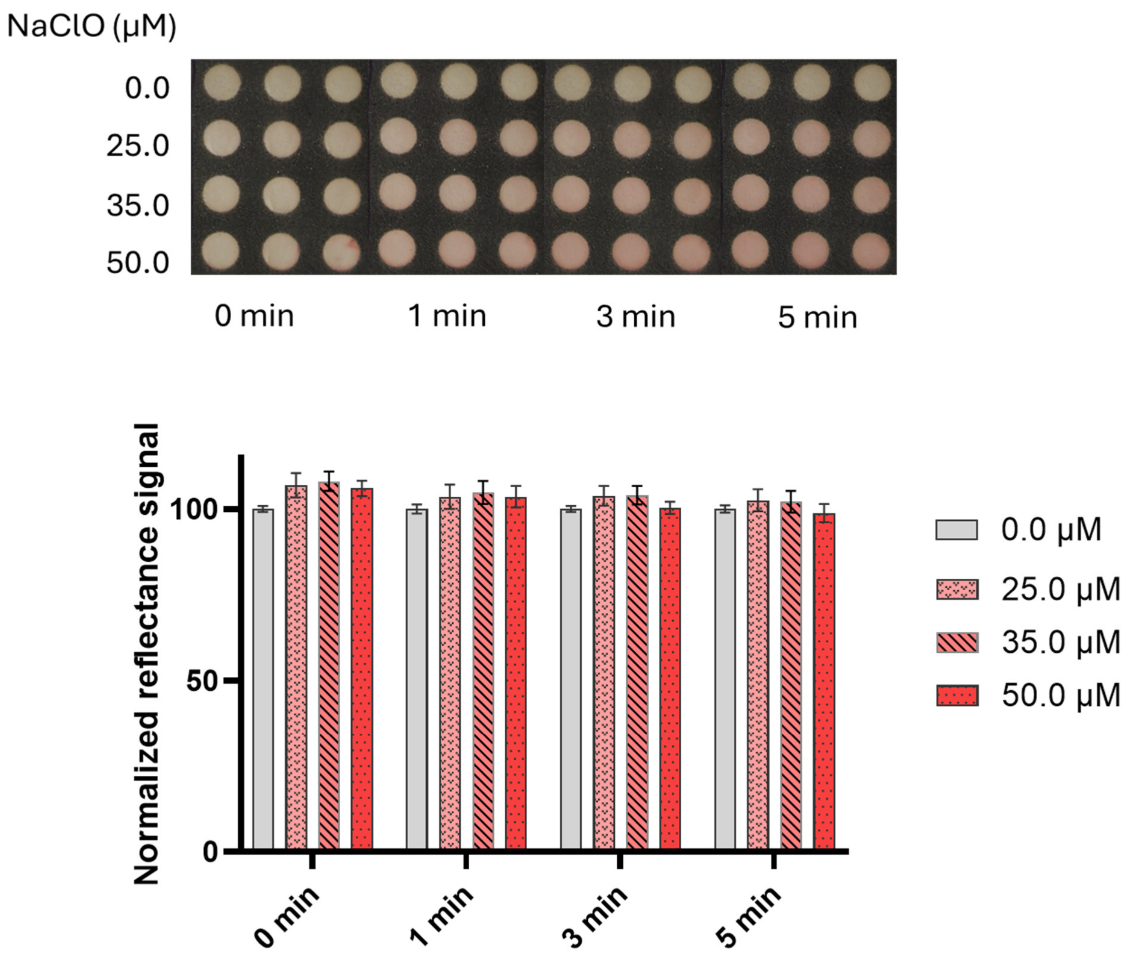

Figure S8: Images of the Chlorine-PAD obtained by drying 40 µL of DPD at room temperature (23 °C) for 24 h in the dark and tested with 15 µL of sample prepared in a 1.0:20.0 ratio (19 µL DB solution + 1 µL NaClO). Reflectance signals were acquired after 0, 1, 3 and 5 minutes in standard room lighting, avoiding shadows, with the Honor 200 PRO smartphone placed at 20 cm vertical distance from the plane of the paper.

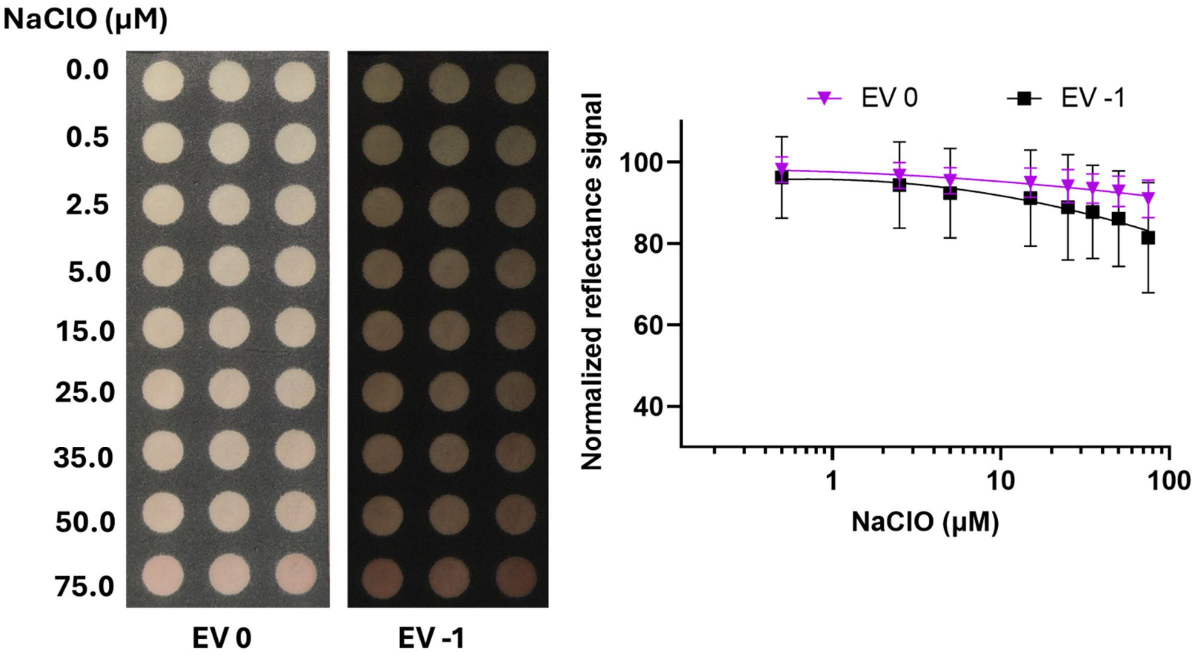

Figure S9: Calibration curve for NaClO obtained using the Chlorine-PAD, obtained by drying 40 µL of DPD at room temperature (23 °C) for 24 h. The test was performed by adding 15 µL of sample prepared in a 1.0:5.0 ratio (20 µL DB solution + 5 µL NaClO). Reflectance signals were acquired in standard room lighting, avoiding shadows, using the Honor 200 PRO smartphone with EV 0 and EV -1, placed at 20 cm vertical distance from the plane of the paper.

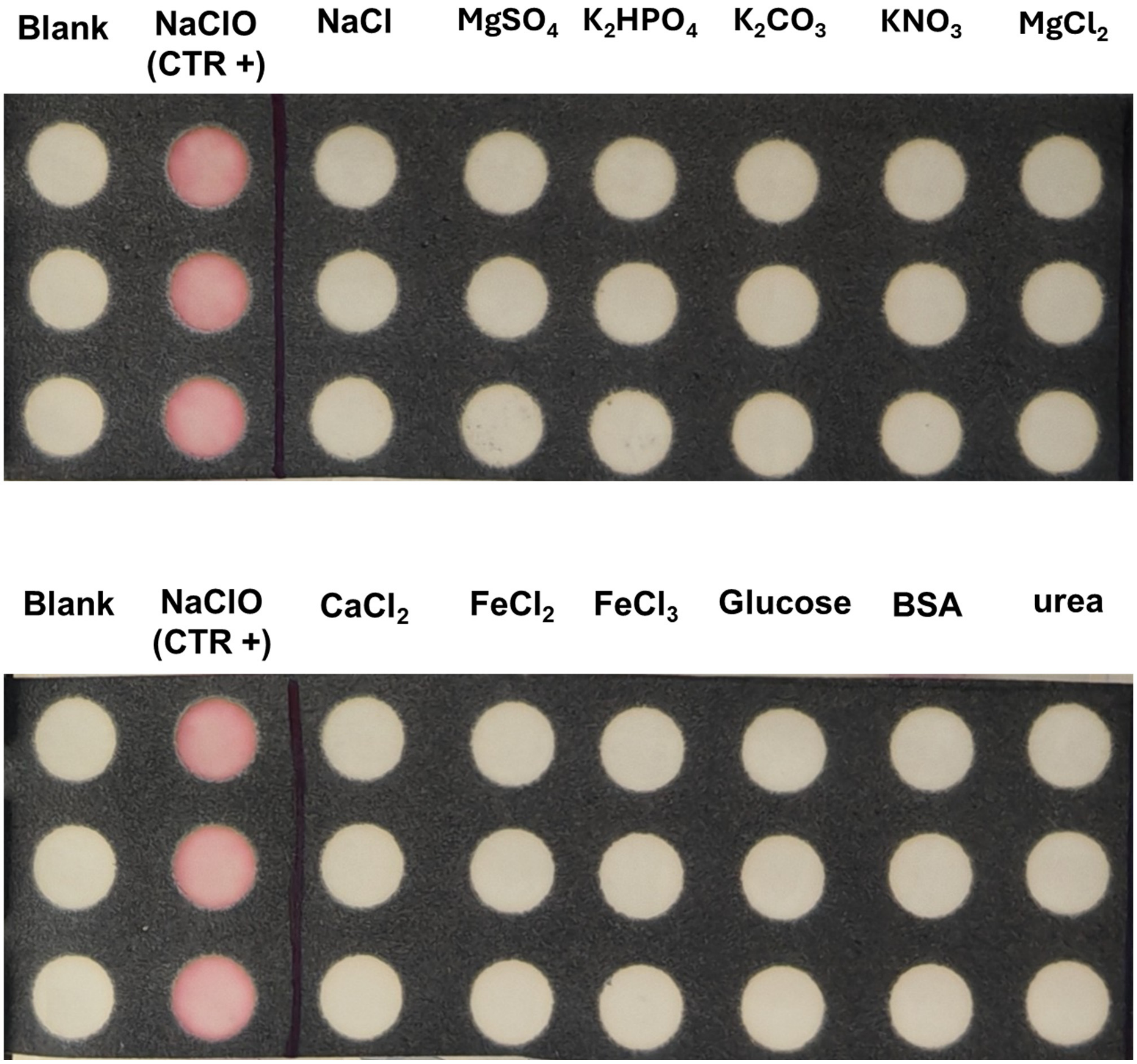

Figure S10: Selectivity studies of the Chlorine-PAD with different interferents (35 µM) compared to NaClO 35 µM (CTR+). Reflectance signals are normalized to the CTR(-).
